# 69 Long-Term Sustained Reduction in Blood Culture Utilization Following Crisis-Driven Stewardship

**DOI:** 10.1017/ash.2026.10499

**Published:** 2026-06-23

**Authors:** Laurel Legenza, Yu Chin Huang, Song Gao, Mike Hasinoff, Taylor Wahlig, Maria Sundaram, Thomas Fritsche, Sanjay Shukla, Josh Petrie

**Affiliations:** 1 University of Wisconsin-Madison School of Nursing; 2 University of WIsconsin-Madison; 3 Marshfield Clinic Research Institute

## Abstract

**Background:** Our prior research demonstrated that Escherichia coli antimicrobial resistance varies geographically at a neighborhood level and antibiotic resistance risk is associated with patient demographics. This study aimed to examine whether antimicrobial resistance exhibits spatial dependence beyond individual-level risk factors. **Methods:** We performed retrospective geospatial analyses of patient characteristics and 2019-2024 E. coli antibiotic susceptibilities from Marshfield Clinic. Patients who had cultures positive for E. coli with susceptibility data were included with their first unique isolate per year (N = 36,043). All covariates were encoded as binary (0/1) indicators, including sex (female), farm residence, public insurance coverage, age under 18 years, age 65 years or older, White race, and antibiotic exposure within the prior 90 days. Outcomes included binary indicators of E. coli susceptibility vs non-susceptibility (intermediate or resistant) to ciprofloxacin (CIP), trimethoprim/sulfamethoxazole (SXT), cefazolin (CFZ), ceftriaxone (CRO), nitrofurantoin (FM), and tetracycline (TE). We first estimated standard logistic regression (binomial logit) models with antimicrobial non-susceptibility as the dependent variable and the full set of covariates as predictors. We then estimated spatial autoregressive probit models using Bayesian Markov Chain Monte Carlo methods (SAR-probit), which explicitly incorporates spatial dependence through a spatially lagged latent propensity. Spatial relationships were encoded using a K-Nearest-Neighbors-based spatial weights matrix constructed from point coordinates, with the neighborhood size (k = 25) selected based on a preceding calibration procedure. We compared non-spatial and spatial models to assess whether spatial clustering in antimicrobial resistance persists after accounting for demographic and exposure-related covariates. **Results:** Consistent with our prior results, logistic regression showed many patient demographics as significant independent predictors, with all variables significantly associated with ciprofloxacin resistance (p < 0.05) and some variation by variable with each of the other antibiotics. The spatial autoregressive parameter results indicated risk of E. coli non-susceptibility is influenced by nearby neighborhood non-susceptibility for all analyzed antimicrobials: CIP (rho = 0.633, p < 0.001), SXT (rho = 0.596, p < 0.001), CFZ (rho = 0.603, p < 0.001), CRO (rho = 0.639, p < 0.001), FM (rho = 0.708, p < 0.001), and TE (rho = 0.580, p < 0.001). **Conclusions:** An individual’s risk for an antibiotic-resistant E. coli infection is affected by their neighbors’ antibiotic resistance. This statistically significant geographic pattern in antibiotic resistance remains while accounting for known patient-level risk factors for antibiotic resistance. The results support patient-level strategies that are informed by community antibiotic resistance surveillance.

